# Neurodegeneration and its potential markers in the diagnosing of secondary progressive multiple sclerosis. A review

**DOI:** 10.3389/fnmol.2023.1210091

**Published:** 2023-09-12

**Authors:** Aleksandra Pogoda-Wesołowska, Angela Dziedzic, Karina Maciak, Adam Stȩpień, Marta Dziaduch, Joanna Saluk

**Affiliations:** ^1^Clinic of Neurology, Military Institute of Medicine–National Research Institute, Warsaw, Poland; ^2^Department of General Biochemistry, Faculty of Biology and Environmental Protection, University of Lodz, Lodz, Poland; ^3^Medical Radiology Department of Military Institute of Medicine – National Research Institute, Warsaw, Poland

**Keywords:** neurodegeneration, markers of neurodegeneration, multiple sclerosis, markers in neuroimaging, markers of multiple sclerosis progression

## Abstract

Approximately 70% of relapsing-remitting multiple sclerosis (RRMS) patients will develop secondary progressive multiple sclerosis (SPMS) within 10–15 years. This progression is characterized by a gradual decline in neurological functionality and increasing limitations of daily activities. Growing evidence suggests that both inflammation and neurodegeneration are associated with various pathological processes throughout the development of MS; therefore, to delay disease progression, it is critical to initiate disease-modifying therapy as soon as it is diagnosed. Currently, a diagnosis of SPMS requires a retrospective assessment of physical disability exacerbation, usually over the previous 6–12 months, which results in a delay of up to 3 years. Hence, there is a need to identify reliable and objective biomarkers for predicting and defining SPMS conversion. This review presents current knowledge of such biomarkers in the context of neurodegeneration associated with MS, and SPMS conversion.

## 1. Introduction

Multiple sclerosis (MS) is an inflammatory degenerative disorder of the central nervous system (CNS) that represents an important cause of disability among young adults. It is classified on the basis of clinical phenotype. In most cases, the disorder begins as relapsing-remitting MS (RRMS), in which inflammation in the CNS, driven by adaptive and innate components of the immune system, leads to myelin destruction and rapid neurological deterioration; this manifests as discrete episode of acute neurological dysfunction, followed by complete or partial remission with subsequent resolution of symptoms (Ciccarelli et al., 2014; Abdelhak et al., 2017). However, many patients go on to experience secondary progressive MS (SPMS) within 10–15 years of the onset of RRMS, where disability progresses independently of relapses. In this condition, the acute exacerbations are less frequent, but the patient undergoes a gradual worsening of neurological dysfunction, with or without periods of remission, and may be subject to increased disability and new brain lesions over time (Lublin et al., 2014; Dendrou et al., 2015). In 2020, the global prevalence of MS was estimated to be 2.8 million people, an increase of 0.5 million compared to 2013. The mean age at diagnosis is 32 years, making this disease not only a major health problem but also a socioeconomic challenge (Walton et al., 2020).

Multiple sclerosis is arguably the most important idiopathic demyelinating disease of the spinal cord and brain, characterized by axonal loss and gliosis and progressive neurodegeneration (Ding et al., 2015; Jeong, 2017; Campbell and Mahad, 2018). While inflammatory activity may decrease as the disease progresses, it is supplanted by increasing mitochondrial dysfunction, oxidative stress, intracellular ion imbalance, and inflammatory activation of microglia; these factors exacerbate the degradation of the myelin sheath and progressive axonal loss, and thus the neurodegeneration that predominates in SPMS (Mahad et al., 2015).

While, neuronal loss is restricted in physiological conditions, even in seniors, it represents a fundamental pathological feature in neurodegenerative disease (Thompson et al., 2018b). Such loss can be multifocal and variable in the CNS; MS has a complex pathogenesis influenced by various genetic and environmental factors and exhibits an unpredictable course, and as such, patients experience an individual clinical manifestation (Correale and Gaitán, 2015; Ziemssen et al., 2016; Waubant et al., 2019). Owing to the heterogeneity of clinical manifestations, rate of progression, and response to treatment, reflecting the existence of several various pathogenetic mechanisms, MS still remains difficult to monitor: there is currently no clearly established imaging, immunologic, clinical, or pathologic criteria for establishing the juncture of transition from RRMS to SPMS. Therefore, no standard definition of SPMS has yet been established, despite the existence of many potential ways to monitor disease progression (Lublin et al., 2014).

Although criteria for the diagnosis of MS were published in 2001, and have since been modified and updated based on later evidence and diagnostic methods (McDonald et al., 2001; Polman et al., 2011; Thompson et al., 2018a), no clear official criteria exist for diagnosing and monitoring SPMS progression; there are of crucial importance for providing optimal treatment. The task of formulating such criteria is hampered by the heterogeneity of MS and its complex pathophysiology, characterized by both inflammation and neurodegeneration. From a clinical point of view, inflammatory processes play a key role in the subacute onset of clinical symptoms, with the development of focal lesions in the CNS. However, while, axonal degeneration begins early in active MS (Ferguson et al., 1997; Trapp et al., 1998) this does not initially manifest itself as a disability due to the remarkable ability of the brain to compensate (Bjartmar et al., 2000).

Inflammation exhibits a robust association with demyelination and neurodegeneration across all stages of MS and represents a key factor in the pathogenesis. The degeneration of demyelinated axons has been postulated as the primary feature of SPMS and a substantial contributor to the gradual progression of a patient’s disability (Mahad et al., 2015). In MS, T, and B cells are present in demyelinating lesions, and CNS antigen-specific immune responses were detected in the peripheral blood. In addition, genome-wide association (GWAS) investigating MS has revealed overexpressed genes associated with immune relevance (Beecham et al., 2013; Lassmann, 2013). Inflammation is an integral part of SPMS. Both SPMS as in RRMS are characterized by similar levels of B cell and T cell markers in the cerebrospinal fluid (CSF) (Komori et al., 2015). CD4+ and CD8+ T cells are widely distributed throughout the parenchyma, whereas B cells predominantly aggregate in the meninges and perivascular cuffs (Luchetti et al., 2018). Recent studies indicate that the genes associated with T-helper type 1 (Th1)/Th17-mediated microglia activation, oxidative stress, DNA damage, and cell death play roles in demyelination and neurodegeneration (Fischer et al., 2013). Peripheral activation and subsequent migration of autoreactive Th1 and Th17 cells into the CNS is widely considered to be a critical step in MS pathogenesis (Romme Christensen et al., 2013).

Conversely, there is growing data that neurodegeneration might not only result from the inflammation process but that it may also initiate the pathological cascade resulting in MS (Baxi et al., 2015; Scheld et al., 2016). Published studies suggest that oligodendrocyte apoptosis, axonal damage, microglial activation, and subsequent neurodegeneration could initiate immune cell recruitment into the CNS and lesions development. Hence, inflammation in MS might be a second step during the formation of novel inflammatory lesions and demyelination, leading to oligodendrocyte apoptosis and, ultimately, neurodegeneration (Scheld et al., 2016).

Reactive astrogliosis is a prevalent occurrence within MS lesions and results in the ongoing generation of pro-inflammatory factors, including tumor necrosis factor (TNF), reactive oxygen species (ROS), and reactive nitrogen species (RNS) (Linnerbauer et al., 2020). The majority of patients with RRMS develop SPMS, an age-dependent clinical milestone typically occurring at 45 years of age, irrespective of RRMS onset age. SPMS is associated with augmented cortical demyelination and inflammation of the meninges in contrast to RRMS (Fransen et al., 2020). Throughout disease progression, demyelination and neurodegeneration are associated with activated microglial cells, being induced by diffusible pro-inflammatory factors generated in meningeal follicles and/or the periphery (Romme Christensen et al., 2013). The meningeal follicles comprise follicular dendritic cells and germinal centers of T and B cells. Furthermore, neurodegeneration also can be caused disturbances in axonal ion homeostasis, mitochondrial integrity compromise, and intracellular iron accumulation collectively exert (Singh et al., 2013; Friese et al., 2014). In SPMS, lymphocytes tend to be disseminated within the CNS parenchyma rather than accumulated around a vessel, which is more frequent in RRMS (Faissner et al., 2019). Some analysis of transcriptomic profiles of peripheral blood cells suggests that MS progression may also be triggered by Epstein-Barr Virus (EBV) reactivation, and increased CD8+ responses to lytic EBV antigens have been noted in blood samples from MS patient cohorts with higher ratios of female participants (Angelini et al., 2013).

Unlike inflammation, neurodegeneration is associated with a constant progression in disability and a reduction in quality of life. As the disease progress, demyelination gradually dominates over remyelination, thus resulting in the clinical progression of neurological symptoms and greater disability. Therefore, monitoring neuroaxonal loss and functional status is an important goal in managing MS (individual possibility of CNS compensation) (Rovira et al., 2013). However, the clinical measures of disability applied in routine clinical practice as part of the widely-used Expanded Disability Status Scale (EDSS) are not very sensitive. While the scale mainly estimates the ability to move, it, unfortunately, provides no information about the clinical symptoms affecting the upper limbs or cognitive functioning (Grand’Maison et al., 2018).

Patients with SPMS exhibit a wide range of both physical and mental symptoms that can limit their activity and worsen their disability. In addition, neurological symptoms typically vary from day to day, which can be an obstacle to objectively detecting new symptoms or progression. Therefore, neurologists typically observe a case for at least 6–12 months of distinct progression, before assigning the term “secondary progressive.” Unfortunately, this can result in a significant delay of about 3–4 years in the diagnosis of SPMS, before which, the patients are still considered RRMS (Lublin et al., 2014; Lorscheider et al., 2016). As such, the diagnosis of SPMS defined by Lorscheider et al. (2016) is still regarded as the most accurate (Adamczyk-Sowa et al., 2020). According to this definition, SPMS is characterized by a one-degree progression on the EDSS scale in patients with an initial EDSS score of ≤5.5, or a 0.5-degree increase for patients with an initial EDSS score of ≥6 without experiencing any relapses. Additionally, a minimum EDSS score of 4 is required to diagnose SPMS (Adamczyk-Sowa et al., 2020).

This article summarizes the processes associated with neurodegeneration in MS and then reviews the markers currently believed to be indicative of neurodegeneration in SPMS.

## 2. Potential biochemical neurodegeneration markers associated with SPMS

In recent years, modern methods of molecular biology have driven research on various disease biomarkers, particularly those demonstrating high sensitivity, specificity, repeatability, and non-invasiveness of acquisition. As with many diseases, appropriately-defined markers can help diagnose, predict, and monitor disease activity, as well as the progress of therapy across multiple individual courses of MS. However, the implementation of suitable markers in clinical practice presents a challenge due to the heterogeneity and variable course of the disease.

This review highlights the value of both potential and existing markers in the development of diagnostic strategies for SPMS. The creation of an appropriate algorithm based on such markers will help develop personalized medicine approaches for MS, despite its multiparametric nature. The levels of biochemical markers of neurodegeneration in biological fluids of MS patients are presented in Table 1.

**TABLE 1:** Summary of the results of studies on the level of biochemical markers of neurodegeneration measured in biological fluids of MS patients.

Marker	Study group	EDSS MEDIAN (range) OR (mean ± SD)	DMTs	Materials	Methods	Principal results	Refences
**NfL**	Baseline/5-years follow-up/10-years follow-up: RRMS *n* = 35/29/21 SPMS *n* = 7/12/12 PPMS *n* = 2/2/4	Baseline/5-years follow-up/10-years follow-up: 3.5 (2.0)/3.5 (2.5)/3.5 (4.0)	Baseline/5-years follow-up/10-years follow-up: - interferons *n* = 6/12/9 - glatiramer acetate *n* = 1/5/5 - mitoxantrone *n* = 0/1/1 - natalizumab *n* = 0/0/4 - fingolimod *n* = 0/0/1	CSF	ELISA	- Patients with disability progression demonstrated a significant trend for higher baseline CSF-NfL levels than patients with no disability progression after 5 years; - patients who transformed from RRMS to SPMS at 5 years had a statistically significant higher median CSF-NfL level; - at the time of diagnosis, NfL appears to be an early predictor of long-term clinical outcomes and a biomarker of conversion of RRMS to SPMS	Bhan et al., 2018
	RRMS *n* = 21 SPMS *n* = 43 PPMS *n* = 27 CTR *n* = 10	Baseline/last follow-up: RRMS: 1.5 (1.0–1.5)/1.5 (0–2.0) PP/SPMS: 6.5 (5.5–7.0)/6.5 (6.0–7.5) CTR: NA	No steroid treatment in the previous month and no immunomodulatory/ immunosuppressive treatment in the preceding 6 months.	Plasma	Simoa technology, (Quanterix Corp.)	- Significantly higher concentrations of NfL in PP/SPMS compared to RRMS patients and correlated with EDSS and MSSS (MS Severity Score); - increase of plasma NfL levels in PP/SPMS patients in a longitudinal sub-study; - no association between plasma NfL with prior/subsequent disability progression, as measured by EDSS	Ferraro et al., 2020
	CIS *n* = 11 RRMS *n* = 178 SPMS *n* = 54 PPMS *n* = 14 CTR *n* = 259	MS: 3.0 (2.0–4.0) CTR: NA	At baseline: - untreated *n* = 91 - interferon beta 1b *n* = 53 - interferon beta 1a sc *n* = 40 - glatiramer acetate *n* = 33 - interferon beta 1a im *n* = 24 - mitoxantrone *n* = 7 - azathioprine *n* = 6 - dimethyl-fumarate *n* = 1 - combination therapy of interferon beta 1b and azathioprine *n* = 2	Serum	Simoa technology, (Quanterix Corp.)	- Serum NfL was correlated with concurrent and future clinical parameters of disease activity and severity and MRI; - serum Nfl was associated with brain and spinal cord volume loss; - NfL was suggested conceptually more comprehensive marker than brain MRI	Barro et al., 2018
**NfM**	MS *n* = 47 CDEG (neurodegenerative diseases) *n* = 14 CD (patients with various unrelated neurological diseases – control diseased group) *n* = 21 CTR *n* = 16	3.0 (0.0–6.5) NA NA NA	In 32 patients included immunosuppressive therapy alone or combination of immunosuppressive therapy with immunomodulatory agents. Others had received no treatment prior to the lumbar puncture	Serum CSF	ELISA	- In MS patients, anti-NfL, anti-NfM and anti-TUB levels correlated with each other in the CSF and in the serum and those calculated as intrathecal synthesis; - the highest correlation between anti-NFM and anti-NFL was found in the MS group	Fialová et al., 2009
**NfH**	RRMS *n* = 24 CTR *n* = 11	<5.5 NA	Drug-naive patients	Serum	ELISA	- pNfH level was significantly correlated with EDSS	Herrera et al., 2019
	CIS *n* = 67 NC (controls with neuropsychiatric diseases of non-inflammatory etiology) *n* = 18	1.0 (0.0–2.0) NA	None at the time of the lumbar puncture	CSF	ELISA	- NfH was significantly higher in CIS than in NC; - NFH levels were correlated with physical disability and with change in brain volume over 1 year of follow-up; - NfH levels were not correlated with change in T2 lesion load	Khalil et al., 2013
	CIS *n* = 36 RRMS *n* = 36 CTR *n* = 10	3.0 (1.0–7.0) 5.0 (2.0–8.0) NA	None within at least 6 months prior to recruitment	Plasma	ELISA	- Positive correlation between pNfH and BBB permeability, clinical severity of the disease, index of disease progression, and demyelinated brain lesion volume in the RRMS group	Ljubisavljevic et al., 2016
**GFAP**	RRMS *n* = 15 SPMS *n* = 10 CTR *n* = 28	2.75 (2.6 ± 0.9) 5.5. (5.5. ± 2.6) NA	No patients were on DMT at the first examination. At the second examination: RRMS: -interferon beta *n* = 5 -glatiramer acetate *n* = 1 SPMS: -mitoxantrone *n* = 1.	CSF	ELISA	-GFAP CSF level was correlated with age in each group; -MS patients had increased GFAP levels compared with controls; -GFAP levels were correlated with neurological disability (EDSS) and disease progression (MSSS); -SPMS patients had two-times higher GFAP level in CSF.	Axelsson et al., 2011
	RRMS *n* = 111 PPMS *n* = 18	1.0 (0.0–3.0) 3.5 (2.0–6.0)	48.7% of RRMS on DMT. None from PPMS were on DMT at the first examination.	CSF/Serum	Simoa technology, (Quanterix Corp.)	-Increased level of GFAP in PPMS in serum compared to RRMS; -serum GFAP level was correlated with age, disease duration and disease type; -in RRMS group, the presence of a recent relapse was correlated with lower serum GFAP; -serum GFAP level was correlated with white matter lesion load and inversely correlated with white matter, grey matter and cortical grey matter volumes.	Ayrignac et al., 2020
	RRMS *n* = 46 SPMS *n* = 33 CTR *n* = 13	2.5 (0.0–5.0) 6.0 (2.0–8.0) NA	A total of 40 RRMS (85.11%) and 11 SPMS (33.33%) patients were on DMT: natalizumab *n* = 10; interferon beta-1a *n* = 8; glatiramer acetate *n* = 6; rituximab *n* = 5; dimethyl fumarate *n* = 5; fingolimod *n* = 14; teriflunomide *n* = 3.	Serum	Simoa technology, (Quanterix Corp.)	-Higher serum concentration of GFAP was associated with higher EDSS, older age, longer disease duration, progressive disease course and MRI pathology.	Högel et al., 2020
**Cf-mtDNA**	Newly diagnosed RRMS *n* = 21 CTR *n* = 23	No data.	None from PPMS were on DMT at the first examination.	CSF	qPCR (determination of mtDNA copy number); ELISA (for NfL determination)	-Increased amount of cf-mtDNA in the CSF in RRMS compared to control group; -RRMS patients had higher NfL level in CSF compared to healthy donors; -the levels of mtDNA and disease duration were inversed correlated; -mtDNA may provide a stage-specific marker for disease activity.	Varhaug et al., 2017
	Post-mortem analysis: progressive MS (PMS) *n* = 36 CTR *n* = 43	No data	No data	CSF	qPCR (determination of mtDNA copy number and identification of deletion regions)	-Diminished cf-mtDNA copies/μl in CSF from PMS patients.	Lowes et al., 2019
	RRMS *n* = 50 SPMS *n* = 27 PPMS *n* = 13 NINDC (non-inflammatory neurologic disease control) *n* = 23 INDC (inflammatory neurologic disease control)_*n* = 7	3.0 (2.5–4.0) 6.0 (4.0–7.0) 4.0 (3.5–6.0) NA NA	Ratio DMT: ODMT (no use of DMT): -RRMS: 19:31 -SPMS: 4:23 -PPMS: 0:0	CSF	Droplet digital polymerase chain reaction (ddPCR)	-PMS patients showed a significant increased concentration of cf-mtDNA in the CSF compared to NINDC; -patients with RRMS showed a trend toward lower concentrations of mtDNA compared to SPMS patients; -patients with a high T2 lesion volume displayed higher mtDNA concentrations compared to patients with a relative low T2 lesion volume.	Leurs et al., 2018
**sTREM2**	RRMS *n* = 36 SPMS *n* = 20 PPMS *n* = 3 CTR *n* = 27	3.0 (1.8–4.0) 6.0 (4.5–6.5) 6.5 (6.3–7.5) NA	RRMS: -natalizumab *n* = 27 -mitoxantrone *n* = 1 SPMS: -mitoxantrone *n* = 6 PPMS: -mitoxantrone *n* = 2	CSF	ELISA	-CSF levels of sTREM 2 were significantly increased in patients with RRMS, SPMS, and PPMS compared with controls; -the levels of sTREM 2 were reduced after mitoxantrone treatment.	Öhrfelt et al., 2016
	RRMS *n* = 52 PPMS *n* = 21 OIND (other inflammatory neurologic diseases) *n* = 27 NIND (non-inflammatory neurologic diseases) *n* = 41	3.5 (1.0–6.5) 6.0 (2.0–9.0) NA NA	RRMS *n* = 26 PPMS *n* = 0	Blood CSF	Flow cytometry ELISA	-Compared to NIND subjects, CSF sTREM 2 levels were significantly higher in RRMS and PPMS subjects, as well as in OIND subjects; -levels of sTREM 2 in blood did not differ among the groups; -sTREM 2 was highly expressed on myelin-laden macrophages in 8 active demyelinating lesions from 4 autopsied MS subjects.	Piccio et al., 2008
	CSF sample: CIS *n* = 9 RRMS *n* = 10 SPMS *n* = 10 PPMS *n* = 13 OND (other neurological diseases) *n* = 15 Serum sample: CIS *n* = 23 RRMS *n* = 55 SPMS *n* = 44 PPMS *n* = 42 OND (other neurological diseases) *n* = 87 CTR *n* = 62	2.0 (1.0–2.6) 3.0 (1.0–5.8) 6.5 (5.3–7.0) 6.5 (6.0–6.9) NA 1.0 (0.0–2.0) 2.5 (1.0–4.0) 6.5 (5.5–7.5) 6.5 (6.0–7.4) NA NA	Subjects with CIS or MS had not received corticosteroids or immunomodulatory therapy for at least 3 months prior to blood or CSF sample collection.	CSF/Serum	ELISA	-sTREM 2 was significantly elevated in the CSF, but not in the serum, in MS compared to OND. -the level of sTREM 2 in the CSF is related to measures of T cell activation (sCD27), neuroaxonal damage (NfL and pNfH), disability (EDSS) and disease severity (MSSS).	Ioannides et al., 2021
	RRMS *n* = 51	1.5 (1.0-2.0)	None from RRMS were on DMT at the first examination.	CSF	Bio-Plex multiplex cytokine assay (Bio-Rad lab.)	-The CSF level of sTREM 2 showed significant correlations with inflammatory cytokines IL-8, G-CSF, and IL-5.	Azzolini et al., 2022
**α -syn**	Post-mortem analysis: RRMS *n* = 4 SPMS *n* = 2 PPMS = 2 Unknown MS type *n* = 4 CTR *n* = 6	No data	No data	Autopsied brain samples (cerebral hemispheres and brainstem)	Double-Immunofluores cence Microscopy	-α-syn immunoreactive cells were identified in active (15/15 lesions in the brainstem, 9/13 in cerebral hemispheres) and chronic active (14/15 in the brainstem, 12/22 in cerebral hemispheres) lesions but were absent in chronic inactive lesions (0/31).	Lu et al., 2009
	RRMS *n* = 50 SPMS *n* = 10 CTR *n* = 60	Mean for MS: 2.0 (0.5–6.0)	-Interferon beta 1 a *n* = 23 -interferon beta 1 b *n* = 9 -glatiramer acetate *n* = 5 -fingolimod *n* = 10 -dimethyl fumarate *n* = 6 -teriflunamide *n* = 3 -natalizumab *n* = 4	Serum	ELISA	-Serum α-syn and oligomer α-syn levels were significantly lower in the MS patients compared to the control group; -serum oligomer α-syn/α-syn ratio was higher in the MS patients compared to the control group and in SPMS compared to RRMS, but was not statistically significant; -there was a significant positive correlation between α-syn and oligomer α-syn in MS patients.	Bilge et al., 2021
**CHI3L1**	Experiments: RRMS *n* = 21 CTR = 21 Verification: CIS *n* = 40 RRMS *n* = 38 PMS = 16 CTR *n* = 16	No data	No data	Autopsied brain samples CSF/Serum CSF	Immunostaining ELISA Isobaric mass tag labeling (analysis of CSF proteome)	-CHI3L1 showed a strong expression in brain of MS patients, especially in astrocytes and microglial cells from white matter plaques; - CSF and serum CHI3L1 levels increased with the disease stage; -CIS patients with high CSF and serum CHI3L1 levels converted more rapidly to RRMS; -CSF CHI3L1/CHI3L2 ratio accurately discriminated PMS from RRMS.	Hinsinger et al. (2015)
	RRMS *n* = 99 SPMS *n* = 35 PPMS *n* = 23	2.0 (1.0–3.0) 5.5. (4.25–6.5) 5.0 (3.5–6.0)	- Interferon beta *n* = 26 -glatiramer acetate *n* = 3 -fingolimod *n* = 2 natalizumab *n* = 15 -antiCD20 *n* = 3	CSF	ELISA	-CSF level of CHI3L1 were higher in MS patients compared to healthy control; -high level of CHI3L1 were characteristic of progressive disease; CSF CHI3L1 level was a predictor for 1-point EDSS worsening and progression during follow-up.	Gil-Perotin et al., 2019
**CXCL13**	CIS-RRMS *n* = 18 SPMS *n* = 8 Neurosyphilis (Lues) *n* = 6 Lyme-neuroborreliosis (LNB) *n* = 13 Bacterial meningitis *n* = 10 Viral meningitis *n* = 10 Non-inflammatory neurological diseases controls (NIND) *n* = 10	No data	CIS-RRMS: - interferon beta *n* = 1 - no therapy *n* = 17 SPMS: - mitoxantrone *n* = 3 - rituximab *n* = 1 - no therapy *n* = 4	Serum CSF	Luminex MAGPIX^®^	- CXCL13 was strongly associated with B-cells; - CXCL13 was confirmed as a reliable marker for CSF B-cell recruitment; - CXCL13 may be used as a clinical marker to predict disease activity in MS	Lepennetier et al., 2019
	MS *n* = 67: CIS *n* = 41 RIS *n* = 4 RRMS *n* = 9 SPMS *n* = 1 PPMS *n* = 13 non-inflammatory neurologic disease patients (NIND) *n* = 67	No data	No corticosteroid therapy 30 days before the lumbar puncture.	Serum CSF	Luminex MAGPIX^®^ ELISA	- CXCL13 index was the best predictor of future disease activity in MS patients; - CXCL13 index values were significantly elevated in activity-positive MS patients compared to activity-negative patients; - as a single predictor, CXCL13 index exhibit better performance than both OCBs and CSF NfL in sensitivity, specificity, and positive and negative predictive value, for future disease activity in MS patients; - combining CXCL13 index and CSF NfL status improved sensitivity and predictive values for disease activity in MS patients; - CXCL13 index is an excellent candidate prognostic biomarker for disease activity in patients with MS	DiSano et al., 2020
	RRMS *n* = 25 PPMS *n* = 24 CTR *n* = 31	1.5 (1.0–7.5) 3.75 (1.5–9) NA	None received immunomodulatory and immunosuppressive treatment for the last 12 months or steroids for a minimum of 6 months before sample collection.	Serum CSF	ELISA	- CSF CXCL13 levels was significantly higher in RRMS and PPMS than in the control group; - in the stable phase of disease, CSF CXCL13 levels in RRMS and PPMS patients demonstrated no significant difference; - patient age and CXCL13 levels in the CSF in all groups demonstrated no significant correlation;	Iwanowski et al., 2017
**miRNA**	RRMS *n* = 14 S/PPMS *n* = 11 S/PPMS independent validation group CTR *n* = 11	1.5 ± 1.0 5.3 ± 1.6 6.0 ± 1.1 NA	RRMS: *n* = 6 S/PPMS *n* = 4 S/PPMS independent validation group: *n* = 7	Serum circulating exosomes	Small RNA sequencing	- miR-15b-5p, miR-23a-3p, miR-223-3p, miR-374a-5p, miR-30b-5p, miR-433-3p, miR-485-3p, miR-342-3p, and miR-432-5p distinguished RRMS from PMS - exosomal-associated miRNAs have utility as biomarkers in MS	Ebrahimkhani et al., 2017
	RRMS *n* = 31 SPMS *n* = 15 PPMS *n* = 16 CTR *n* = 15	Acute RRMS: 3.73 ± 1.90 Stable RRMS: 2.25 ± 2.11 SPMS: 6.64 ± 1.40 PPMS: 6.36 ± 1.63 CTR: NA	No treatment for at least 2 months before the blood withdrawal.	Serum	qPCR	- miR-572 can serve as a tool that helps to differentiate PPMS and SPMS as well as distinguishes between relapsing and remitting phase in RRMS - miR-572 may be a non-invasive biomarker for remyelination	Mancuso et al., 2015
	RRMS *n* = 9 SPMS *n* = 9 CTR *n* = 9	1.2 ± 1.2 5.6 ± 1.96 NA	No treatment with glatiramer acetate or interferons in the past 3 months, any other DMTs in the past 6 months, no steroids in the past month.	Ethylenedia minetetraace tic acid plasma	qPCR	- miRNA let-7 family differentiated SPMS from CTRs and RRMS from SPMS	Gandhi et al., 2013
**Cf-mtDNA**	Newly diagnosed RRMS *n* = 21 CTR *n* = 23	No data	None from PPMS were on DMT at the first examination.	CSF	qPCR (determination of mtDNA copy number); ELISA (for NfL determination)	-Increased amount of cf-mtDNA in the CSF in RRMS compared to control group; -RRMS patients had higher NfL level in CSF compared to healthy donors; -the levels of mtDNA and disease duration were inversed correlated; -mtDNA may provide a stage-specific marker for disease activity.	Varhaug et al., 2017
	Post-mortem analysis: progressive MS (PMS) *n* = 36 CTR *n* = 43	No data	No data	CSF	qPCR (determination of mtDNA copy number and identification of deletion regions)	-Diminished cf-mtDNA copies/μl in CSF from PMS patients.	Lowes et al., 2019
	RRMS *n* = 50 SPMS *n* = 27 PPMS *n* = 13 NINDC (non-inflammatory neurologic disease control) *n* = 23 INDC (inflammatory neurologic disease control)_*n* = 7	3.0 (2.5–4.0) 6.0 (4.0–7.0) 4.0 (3.5–6.0) NA NA	Ratio DMT: ODMT (no use of DMT): -RRMS: 19:31 -SPMS: 4:23 -PPMS: 0:0	CSF	Droplet digital polymerase chain reaction (ddPCR)	-PMS patients showed a significant increased concentration of cf-mtDNA in the CSF compared to NINDC; -patients with RRMS showed a trend toward lower concentrations of mtDNA compared to SPMS patients; -patients with a high T2 lesion volume displayed higher mtDNA concentrations compared to patients with a relative low T2 lesion volume.	Leurs et al., 2018

### 2.1. Neurofilament light chain (NfL)

The levels of neurofilaments, an axonal structural protein, particularly neurofilament light chain (NfL), a highly-specific predictor of neuronal cell damage and eventual neuronal cell death, in the CSF and blood rise upon neuroaxonal damage; they are hence gaining increasing attention (Khalil et al., 2018). NfL appears to be a promising candidate for a reliable biomarker of MS development. Although NfL is typically present only in the neuroaxonal compartment, it is released following neuro-axonal damage associated with neurodegeneration processes, and the level in blood correlates with disease severity and/or neurodegenerative disease progression. Blood NfL level was shown to greatly correlate with CSF NfL level in MS patients (Abdelhak et al., 2018).

Neurofilament light chain is the most widely-studied biomarker for monitoring MS disease progression. High NfL levels at diagnosis reflect a higher likelihood of neurodegeneration and a higher risk of conversion from RRMS to SPMS. In addition, CSF NfL levels at the time of diagnosis seem to be an early predictive biomarker of long-term clinical outcomes and the development of SPMS. Bhan et al. (2018) report that patients with RRMS who progressed in EDSS after 5 years had significantly higher CSF levels of NfL than those who did not (Bhan et al., 2018). Similarly, Salzer et al. (2010), indicate that RRMS patients who later developed SPMS had higher CSF NfL levels (Salzer et al., 2010). Moreover, two different studies (Disanto et al., 2017; Barro et al., 2018) found a notable correlation between elevated levels of serum NfL and long-term progression of disability in MS, and between high serum NfL levels and both brain and spinal cord volume loss (Disanto et al., 2017; Barro et al., 2018). Similarly, Högel et al. (2020) found that NfL CSF levels were shown to correlate with EDSS and that NfL levels correlated with advanced age, EDSS, disease duration, and Multiple Sclerosis Severity Score (MSSS) (Högel et al., 2020). In addition, increased plasma NfL was noted in patients with progressive MS, and the level increased over time in patients at follow-up (Ferraro et al., 2020). Gnanapavan et al. (2013a) demonstrated that serum NfL correlates well with brain atrophy, detected by magnetic resonance imaging (MRI), and clinical biomarkers of progression (Gnanapavan et al., 2013a), suggesting that it may be an excellent surrogate marker of neurodegeneration. Finally, Williams et al. (2021) report that while NfL is a valuable biomarker of neuroinflammation and future brain atrophy, and can be used for monitoring immunosuppressive treatment, it does not appear to be suitable for neuroprotective disease-modifying therapies in MS (Williams et al., 2021).

### 2.2. Neurofilament heavy chain (NfH)

Presently, there is little evidence to support the use of neurofilament medium chain (NfM) as a biomarker of neurodegeneration biomarker. Additionally, measuring NfM levels in CSF and blood presents a greater technical challenge compared to NfL and NfH levels (Zucchi et al., 2020). The NfM subunits are highly sensitive to protease after dephosphorylation, suggesting that the phosphate groups in these subunits resist protease separation, and thus increase stability in the Nf cytoskeleton in axons (Yuan et al., 2017); however, NfM overexpression has been found to lead to motor neuron dysfunction and paralysis in mice (Yuan et al., 2017). The deletion of NfM in mice also reduced the diameter of large-caliber axons and induced age-associated atrophy of the motor axons (Elder et al., 1999).

Another study compared serum and CSF levels and intrathecal synthesis of anti-NfL IgG, anti-NfM IgG, and anti-TUB (tubulin) IgG, between MS patients and those with other neurological diseases (Fialová et al., 2009). The findings revealed a consistent antibody spectrum against cytoskeletal proteins in the serum of MS patients, that was not present in the sera of the other neurological patients (Fialová et al., 2009). This is probably due to the contiguous decomposition of neurons and axons associated with defective immune regulation in MS.

### 2.3. Neurofilament heavy chain (NfH)

The neurofilament heavy chain (NfH) is an essential component of the neuronal cytoskeleton. It is highly expressed in large-caliber axons, playing a critical role in maintaining the axonal structural integrity, and can be used to indicate the extent of neuro-axonal damage (Petzold et al., 2016; Herrera et al., 2019). NfH levels in the CSF were related to the clinical severity of MS and neurodegeneration in MRI (Khalil et al., 2013; Petzold et al., 2016). Elevated levels of NfH in the CSF were associated with a higher risk of developing MS in patients with clinically isolated syndrome (CIS), suggesting that NfH may be a useful biomarker for predicting conversion to MS (Khalil et al., 2013). In another study, serum NfH levels were found to be 37 pg/mL in RRMS patients, 67 pg/mL in SPMS patients, and 72 pg/mLin PPMS patients indicating that NfH may be a useful biomarker for predicting the long-term course of the disease (Verberk et al., 2021); in addition, serum NfH level was associated with the number of T2 lesions and the duration of the disease, suggesting that serum-NfH may be useful as a biomarker of MS burden (Verberk et al., 2021).

A cross-sectional biomarker study found plasmatic phosphorylated (p)NfH level to be positively correlated with 8-hydroxy-2′-deoxyguanosine (8-OHdG), gadolinium (Gd)-enhancing lesion, EDSS score, BBB permeability, and disease progression in CIS and RRMS patients (Ljubisavljevic et al., 2016). However, it is important to note that SPMS patients are more likely to be pNfH-positive in serum than RRMS/first demyelinating event patients and that the positive status of pNFH in serum is associated with more severe disease and a higher volume of T2 lesions; this is consistent with the hypothesis that the presence of the protein in serum may reflect a higher level of damage to CNS axons (Gresle et al., 2014).

Promising results were yielded by a recent study indicating that serum pNFH may be a potential biomarker for the assessment of MS disease activity and disability. The study indicated significantly higher levels of pNFH in MS patients than in healthy controls and even higher levels among relapsing than remission groups. With a sensitivity of 95% and specificity of 100%, the results suggest that pNFH has the potential to be a useful prognostic or monitoring tool for MS (Shehab et al., 2019).

Despite promising results, several challenges remain before NfH can be used as a biomarker for MS. For example, NfH levels can fluctuate over time (Gresle et al., 2014) and may be affected by factors such as age, body mass index (BMI), and total blood volume (Revendova et al., 2022). Furthermore, there is currently no standardized method to measure NfH levels, and the different tests achieve inconsistent results (Gnanapavan et al., 2013a; Ljubisavljevic et al., 2016; Khalil et al., 2018; Verberk et al., 2021). NfH is also not specific for MS and can also be elevated in other neurological conditions; however, the combination of NfH with other biomarkers, such as myelin basic protein (MBP) and glial fibrillary acidic protein (GFAP), has been shown to improve the specificity of biomarker panels for the diagnosis and prognosis of MS.

One challenge is the accurate and replicable determination of NfH levels in blood samples. Verberk et al. (2021) note that high-sensitivity tests are required to accurately detect NfH in blood serum, as the use of traditional immunoassays can lead to unreproducible results (Kuhle et al., 2017). Currently, NfL appears to be a better therapeutic biomarker than NfH and is a promising candidate for measuring cerebral axon injury in MS treatment studies (Kuhle et al., 2013).

### 2.4. Glial fibrillary acidic protein (GFAP)

The neuroprotective phenotypes of microglia and astrocytes support neuronal activity, development, and survival due to distinct reactive phenotypes with variable neuroprotective or pro-inflammatory polarity dependent on inflammation or brain injury (Kwon and Koh, 2020; Geladaris et al., 2021). One area of interest in studies of MS neurodegeneration concerns markers of glial activation. One such marker is GFAP, an intermediate cytoskeletal protein predominantly expressed in astrocytes, considered a marker of astroglia activation and astrogliosis (Axelsson et al., 2011). GFAP appears to assume a pivotal role in astrocyte-neuron interactions and cell-cell communication (Shan et al., 2018). Recently, higher serum and CSF GFAP were identified in the progressive phase of MS than in RRMS, and these levels correlated with disease severity, MRI lesion count, and clinical disability in MS (Ayrignac et al., 2020). Moreover, a strong correlation was observed between GFAP and NfL levels (CSF and serum) in progressive MS (Abdelhak et al., 2018). In addition, SPMS patients were found to demonstrate twice the mean annual increase of CSF GFAP (18.9 ng/L) compared to RRMS patients (8.1 ng/L) (Axelsson et al., 2011). Additionally, in patients with progressive MS, higher serum GFAP levels were associated with higher EDSS, older age, and longer disease duration (Högel et al., 2020); these findings indicate that astrogliosis may play an increasing role in the advanced stages of the disease and as such serum GFAP may represent a valuable marker of disease progression.

### 2.5. Soluble triggering receptor expressed on myeloid cells 2 (sTREM2)

Triggering receptor expressed on myeloid cells 2 (TREM2) is a surface receptor predominantly expressed on newly-differentiated and activated macrophages and microglia. TREM2 activation serves as a mediator for eliciting anti-inflammatory responses through the modulating cytokine release, proliferation, migration, and phagocytosis of apoptotic cells and myelin remnants (Colonna, 2023). TREM2 undergoes sequential proteolytic processing by ectodomain detachment, resulting in the release of a soluble form of TREM2 (sTREM2) (Zhong and Chen, 2019); this can accumulate around dead neurons, and pathological protein aggregates, which can be attached to various surface receptors and activate innate immune responses (Song et al., 2018). However, unregulated proteolytic cleavage of TREM2 leads to a heightened concentration of sTREM2, thereby engendering disruption to the BBB (Raha-Chowdhury et al., 2019). This may partially explain the presence of increased CSF levels of sTREM2 in neurodegenerative diseases, such as MS (Piccio et al., 2008; Öhrfelt et al., 2016), amyotrophic lateral sclerosis (Cooper-Knock et al., 2017), Alzheimer’s disease (Rauchmann et al., 2019), and Parkinson’s disease (Woollacott et al., 2018).

In MS, the level of sTREM2 in the CSF has been found to correlate with T cell activation (sCD27; *p* = 0.005), neuroaxonal damage (NfL; *p* = 0.0001 and pNfH; *p* = 0.0006), disability (EDSS; *p* = 0.0079) and disease severity (MSSS; *p* = 0.0006) (Ioannides et al., 2021). In addition, levels of CSF sTREM2 have been identified in patients with MS and other inflammatory neurological diseases (Piccio et al., 2008). CSF sTREM-2 levels were found to be significantly higher in RRMS (*p* = 0.004) and PPMS (*p* < 0.001) subjects, as well as those with other inflammatory neurological diseases (*p* < 0.001), compared to those with non-inflammatory neurological diseases; however, in contrast, no differences in sTREM2 level in whole blood were observed between the patient groups (Piccio et al., 2008). These findings, indicating correlations between the level of sTREM2 in CSF and clinical disability and disease severity in MS, suggest that CSF sTREM2 level might be a serviceable tool for surveillance of disease advancement and evaluation of therapeutic efficacy within the confines of clinical trial settings.

The association between GFAP and sTREM2 and the levels of various inflammatory cytokines suggests crosstalk between CSF inflammation and the activation of astroglia and microglia in MS. Azzolini et al. (2022) indicate positive correlations between CSF GFAP and sTREM2 levels and specific inflammatory molecules suggesting a link between CSF inflammatory levels and astroglial and microglial activation in MS. A relationship between GFAP and sTREM2 and cytokine concentrations could signal immune activation in the CNS. Significant correlations were found between the level of sTREM2 and the concentration of interleukin 5 (IL-5) (*p* = 0.045), IL-8 (*p* = 0.002), granulocyte colony-stimulating factor (G-CSF) (*p* < 0.001), IL-13 (*p* = 0.011), and IL-9 (*p* = 0.001) in CSF. Moreover, a strong positive correlation was found between GFAP and sTREM2 (*p* < 0.00001), and the levels of several inflammatory cytokines *viz*. IL-5 (*p* = 0.045), IL-8 (*p* = 0.002), G-CSF (*p* < 0.001), IL-13 (*p* = 0.011), and IL-9 (*p* = 0.001), suggesting an association between microglial and astroglia activation and CSF inflammation (Azzolini et al., 2022).

Öhrfelt et al. (2016) report that RRMS (*p* < 0.0001), SPMS (*p* < 0.0001), and PPMS (*p* = 0.02) patients demonstrated higher CSF levels of sTREM2 compared to controls, indicating that sTREM-2 could be a promising marker of microglial activation in MS (Öhrfelt et al., 2016). Also, a Mendelian randomization study by Dong et al. (2022) found that a genetic predisposition to higher CSF sTREM2 levels was associated with increased risk of MS (OR = 1.038, 95% CI = 1.014–1.064, *p* = 0.002).

These findings provide further evidence that elevated CSF sTREM2 levels appear to be associated with a higher risk of MS (Dong et al., 2022). As such, sTREM2 has been investigated as a useful marker monitoring macrophage/microglial activation in the brain and disease progression in MS patients.

### 2.6. α-Synuclein (α-syn)

α-Synuclein (α-syn) is a tiny neuronal cytoplasmic protein consisting of 140 amino acids residues encoded by the *SNCA* gene. Although it is predominantly located in neurons, it is also expressed in glia and hematopoietic cells, such as T cells and monocytes/macrophages (Pei and Maitta, 2019). In the human brain, α-syn is localized in the distal reserve pool of synaptic vesicles. Changes in α-syn expression result in disturbances in synaptic transmissions, suggesting that it may play a role in the regulation of neurotransmitter release, brain plasticity, and synaptic function (Burré, 2015). This is in line with research on transgenic mice, in which human α-syn overexpression was associated with impaired synaptic vesicle exocytosis and a reduction in neurotransmitter release (Yavich et al., 2004; Nemani et al., 2010). More specifically, α-syn can shape the function of the immune system, thus participating in T cell development (Shameli et al., 2016). Additionally, it plays a regulatory role in the appropriate processing of antigens within phagocytic and lymphocytic cells through autophagic mechanisms, as evidenced in recent research by Choi et al. (2022).

Although the physiological function of α-syn is still unclear, it is well known to play a critical role in neurodegenerative processes. Under pathological conditions, α-syn undergoes a transformative process, leading to the assembly of deleterious aggregates within cerebral tissue, encompassing oligomeric and fibrillar conformations, thus initiating neurodegeneration (Lashuel et al., 2013). Brown et al. (2022) report that *SNCA* gene ablation improved learning and memory in spite of enhanced plaque burden; interestingly, the treatment slowed the development of cognitive deficits in male mice but exacerbated them in female animals, indicating that the critical role of α-syn on cognitive function is sex-specific (Brown et al., 2022).

The abnormal aggregation of α-syn in neurons or glia has been associated with several neurodegenerative diseases (Bennett, 2005). In MS, α-syn has been studied directly in CNS lesions and CSF. Differences in CSF α-syn levels have been reported between patients with MS and control subjects (Wang et al., 2012; Antonelou et al., 2015). For example, α-syn CSF level was found to be augmented in active MS lesions in postmortem brain tissue, but absent in chronic inactive lesions and control samples (Lu et al., 2009). An increase in CSF α-syn level, observed during acute relapse of MS patients may suggest injury to the axons around the inflammatory lesion and subsequent degeneration. Furthermore, CSF α-syn level was positively correlated with EDSS (*p* < 0.001) (Lu et al., 2009). In contrast, Bilge et al. (2021) report that serum α-syn and oligomeric α-syn levels were remarkably reduced in MS patients compared to control. The presence of low levels of α-syn in MS patients may result from neuronal and synaptic loss, possibly driving neuroinflammation (Bilge et al., 2021). Noteworthy, these results were furtherly discussed by Niu and Li (2021), pointing out the need to verify these studies on a greater population and to collect more data.

The more recent study found that the levels of α-syn in both PBMCs and serum were reduced in patients with MS compared to the control group, and correlated with the severity of the disease, as measured by the EDSS score. Additionally, α-syn was found to be correlated with IL-6 levels and the age of the onset of MS. The discrimination ability of α-syn profiles was demonstrated through ROC curves, indicating its potential clinical utility (Razia et al., 2023). Furthermore, it is observed that mice with α-syn knockout (α-syn-/- mice) are more likely to experience an earlier onset of MS symptoms accompanied by enhanced spinal cord infiltration of CD4+ T cells, mainly of interferon(INF)-γ-producing Th1 cells (*p* < 0.05) and decreased infiltration of regulatory T cells (Tregs) (*p* < 0.01) (Ettle et al., 2016). These findings suggest that α-syn deficiency facilitates the activation of Th1 cells and negatively impacts Tregs, consequently, thereby intensifying the immune response orchestrated by Th1 cells in EAE. In addition, increased α-syn expression in mouse macrophages and microglia resulted in impairments in autophagy, and subsequent dysfunction in phagocytosis and cytokine release (Gardai et al., 2013). Moreover, the effect of α-syn deficiency on CNS degeneration and regeneration was evaluated in two demyelinating animal models: chronic MOG_35–55_-induced EAE and the cuprizone-induced model. Histopathological analysis after EAE induction demonstrated a significantly reduced CNS inflammation and myelin loss in α-syn-deficient mice during late-stage inflammatory demyelination. However, α-syn deficiency did not demonstrate an impact on myelination or neuroinflammatory patterns in the cuprizone-induced model. Based on these findings, it was suggested that α-syn may serve as a regulator of the peripheral immune response during neuroinflammation, thus influencing degeneration in the advanced demyelinating condition (Kuhbandner et al., 2020).

Recently, it was reported that CSF α-syn levels may reflect impaired mitochondrial function (Park et al., 2020) and neuronal apoptosis (Ganjam et al., 2019). It has been found that α-syn participates in mitochondrial activities, enhancing adenosine triphosphate (ATP) synthase efficiency, and reversibly blocking voltage-dependent anion channels, thus controlling the passage of most metabolites in and out of mitochondria, and protecting cells from harmful free radicals (Ludtmann et al., 2018). However, mitochondrial dysfunction is known to be a key feature of α-syn toxicity in neurons and glia. In short, dysfunctional mitochondria lose membrane potential, resulting in increased ROS levels and promoting neurodegeneration. Furthermore, the presence of high levels of free radicals in the environment promotes cell organelle dysfunction and post-translational modification of α-syn, i.e., phosphorylation of serine 129 (Anderson et al., 2006), nitration (Giasson et al., 2000) or ubiquitination (Tofaris et al., 2003), thereby facilitating the formation of toxic oligomers. Oxidative stress plays a key role in the harmful effects of α-syn on neurons with oxidative stress generating an increase in α-syn levels and vice versa. Thus, the combination of abnormal α-syn accumulation together with the influence of oxidative stress may contribute to neuronal death in MS (Luk, 2019).

These findings indicate that the functions of α-syn and its role in the pathology of MS are only partially understood. Nevertheless, evidence suggests that the level of α-syn in biological fluids may be a useful biomarker for patients with MS; however, more evidence concerning the effect of α-syn level on MS development is needed.

### 2.7. Chitinase 3-like 1 (CHI3L1)

Astrocyte-derived chitinase 3-like 1 (CHI3L1) has been found to have a prognostic role in the early phases of MS. High CSF CHI3L1 levels are a risk factor for conversion to MS independently of the presence of powerful predictors of conversion to MS, including IgG oligoclonal bands (OCBs) and brain MRI abnormalities. More importantly, elevated CSF CHI3L1 levels were found to be the only significant independent risk factor associated with the development of disability based on multivariate Cox regression models. In addition, high CHI3L1 levels were also associated with earlier progression to EDSS 3 and EDSS 6, thus confirming that CHI3L1 plays a role in neurological disability development (Comabella et al., 2021). CSF and serum CHI3L1 levels may serve as biomarkers of the early disease stages of MS (CIS). CHI3L1 concentration was found to gradually increase in serum and CSF samples during disease progression, with these levels already being significant in CIS patients. In contrary to CHI3L2, CHI3L1 was found to be meaningfully enhanced in the CSF of patients with progressive stage of MS in comparison to RRMS patients, and elevated CSF CHI3L1 levels were identified as an independent predictor of a worsening disability in patients with MS (Gil-Perotin et al., 2019).

### 2.8. Chemokine (C-X-C motif) ligand 13 (CXCL13)

Probably the most widely-documented chemokine in the pathophysiology of MS is chemokine (C-X-C motif) ligand 13 (CXCL13), a “B-cell attracting chemokine” (Ragheb et al., 2011) known to play an essential role in the recruitment and activation of immune cells to inflammatory *foci* in Pan et al. (2022). CSF CXCL13 and B cell levels were found to increase simultaneously under intact BBB conditions (Lepennetier et al., 2019). Furthermore, CXCL13 levels have been found to correlate with disease severity measured using the EDSS scale, as well as T2 lesions, intrathecal immunoglobulin production, oligoclonal bands (OCBs) and lymphocytes, indicating that it can be used as a prognostic marker in MS (Krumbholz et al., 2006; Khademi et al., 2011; Axelsson et al., 2013; Lindén et al., 2013; Ferraro et al., 2015; Håkansson et al., 2018).

Novakova et al. (2020) report increased levels of NfL and CXCL13 in patients with ongoing disease activity and that a significant proportion of patients without ongoing disease activity still showed elevated levels of these markers in the CSF, indicating residual disease activity. The findings suggest that these biomarkers could potentially exhibit a heightened sensitivity to disease activity in comparison to conventional clinical and MRI metrics (Novakova et al., 2020).

A systematic review and meta-analysis by Bai et al. (2019) clarified a panel of cytokines that exhibit great potential to be used as biomarkers for MS. It was found that CXCL13 was consistently increased in both blood and CSF samples, thus allowing MS patients to be distinguished from healthy donors, and for disease progression to be evaluated (Bai et al., 2019). A more recent study including patients with the radiologically isolated syndrome (RIS), CIS, RRMS, and progressive MS found that CSF/serum CXCL13 may serve as a single predictor in MS; indeed CXCL13 can be considered even more sensitive, specific, and predictive than more well-known OCB and NfL (DiSano et al., 2020).

It was also found that by combining the CXCL13 index with the CSF NfL score, predictive values can be further maximized (Pachner, 2022). Among the 67 patients with MS, 41 demonstrated an initial clinical demyelinating event (ICDE), which is an early signal to apply immunomodulatory therapy. The negative predictive value of intrathecal CXCL13 was 91% in patients with ICDE, indicating that intrathecal CXCL13 accurately predicted the absence of future inflammatory activity in this group of patients. By comparison, the negative predictive value for OCBs was 64% (Pachner, 2022). CXCL13 hence represents a better candidate for employing individually-shaped immunomodulatory therapies in MS patients, based on the anticipated activity of the disease.

In contrast, a recent study found CSF CXCL13 does not predict disease activity, measured by EDSS and MRI, in CIS/RRMS (Hradilek et al., 2023). In addition, it was also found that while CSF CXCL13 levels differed significantly between the MS and control groups in another study, no such differences were found between the RRMS and PPMS group in the stable phase of MS without relapse, despite these two subtypes of MS representing different stages of neurodegeneration (Iwanowski et al., 2017). Otherwise, CXCL13 has been found to increase significantly in MS patients compared to a CIS group (Khademi et al., 2011). Such inconsistencies may be due to some studies not employing optimal analytical techniques; while most studies have been based on ELISA, more accurate results have been achieved using Luminex, SIMOA or immuno-PCR have been proven to provide more sensitive results in CXCL13 detection (Pachner, 2022).

Hence, CXCL13 has great potential to be developed as clinically-certified assays for the diagnosis of MS; however, it should be noted that intrathecal production of CXCL13 is not exclusively characteristic of MS neuroinflammation and may also be associated with other neuroinflammatory diseases (Alvarez et al., 2013; Irani, 2016). Consequently, the CXCL13 index has limited use as a diagnostic biomarker of demyelination diseases.

### 2.9. miRNA

Over the past few years, many papers have examined the changes in microRNA (miRNA) profiles occurring during the different phases of MS (Martinez and Peplow, 2020; Zhou et al., 2020). MiRNAs are short (19–25 nucleotides), non-coding, single-strand RNA sequences that play a pivotal role in gene expression regulation (Broughton et al., 2016).

Several miRNAs are believed to offer potential as neurodegenerative markers. In addition, their expression in different MS subtypes can be studied using less invasive methods than CSF collection, such as plasma or serum samples.

It was found that serum miR-27a-3p expression was significantly upregulated in the RRMS compared to SPMS, with an area under the curve value of 0.7 (Regev et al., 2016). It was pointed out that this miRNA can target genes connected to the neurotrophin signaling pathway, indicated by the Kyoto Encyclopedia of Genes and Genomes (KEGG) database; as such, it may be an early marker of neurodegeneration (Regev et al., 2016). In addition, KEGG pathway analysis found the expression of miR-30b-5p to differ between RRMS and SPMS, and this too is believed to be engaged in modulating neurodegeneration (Ebrahimkhani et al., 2017); it may target, for instance, the Wnt pathway involved in oligodendrocyte development and the remyelination process (Xie et al., 2014; Brennan et al., 2019).

Two other important candidates for neurodegeneration markers are miR-145-5p and miR-572, which are both involved in CNS repair mechanisms. One of the targets for miR-145-5p is myelin gene regulatory factor (MYRF): a transcriptional regulator required for CNS myelination and oligodendrocyte maturation. Kornfeld et al. (2021) identified severe deficits in myelin gene expression and thus neurological abnormalities, in mice lacking MYRF (Kornfeld et al., 2021). It has been suggested that miR-572 may target the neural cell adhesion molecule (NCAM) involved in neuronal sprouting and synaptic remodeling and polysialylated NCAM may be engaged in negative regulation of myelination (Jørgensen, 1995; Charles et al., 2000; Mancuso et al., 2015). In addition, abnormal levels of soluble NCAM in MS CSF may be associated with the progression of the disease, as its levels were found to be reduced in SPMS compared to RRMS (*p* < 0.05) (Gnanapavan et al., 2013b).

In MS, neurodegeneration is strictly associated with the transition from the RRMS to the SPMS phase. Therefore, the markers that distinguish these two pathological subtypes are of great interest to researchers. However, a recent extensive review of the miRNA molecules expression in RRMS and SPMS highlighted that there is still insufficient data indicating that specific miRNAs may be expressed differentially between the particular disease stages (Pietrasik et al., 2021). The most widely studied miRNA in the context of MS pathophysiology is miR-181c-5p, which participates in the regulation of neuronal maturation and synaptogenesis in the brain cortex. CSF miR-181c-5p level appears to be downregulated in SPMS (Haghikia et al., 2012) compared to RRMS; however, this was not confirmed in an independent study (Kramer et al., 2019). Other miRNAs found to be down-regulated in SPMS compared to RRMS include miR-633-5p (Haghikia et al., 2012), miR-27a-3p (Regev et al., 2016), miR-337-3p (Regev et al., 2018), miR-92a-1-3p, miR-145-5p, let-7c and let-7d (Gandhi et al., 2013). One of the most widely-documented miRNAs in the context of MS pathophysiology is miR-155, whose overexpression is accompanied by the promotion of amyloid β (Aβ) production. Since, Aβ is the activator of nuclear factor kappa B (NF-κB) in neurons and astrocytes, excessive production of miR-155 leads to neuroinflammation. Studies also revealed an association between miR-155 and α-synuclein, another marker of neurodegeneration (Rastegar-Moghaddam et al., 2023).

A recent cross-sectional study found patients with PPMS to demonstrate a specific profile of dysregulated circulating CSF miRNAs compared to other individuals with neurological disease, including the signature of let-7b-5p and miR-143-3p. Furthermore, several dysregulated miRNAs in serum (miR-20a-5p, miR-320b, miR-26a-5p, miR-485-3p, miR-142-5p), have been identified as valuable candidates for further study regarding their actual role in MS (Muñoz-San Martín et al., 2023).

### 2.10. Cell-free mitochondrial DNA (cf-mtDNA)

A recent study confirmed the presence of cell-free mitochondrial DNA (cf-mtDNA) in the CSF of patients with Alzheimer’s and Parkinson’s disease, suggesting mtDNA might be a marker for neurodegeneration (Podlesniy et al., 2013; Pyle et al., 2015). Other studies of cf-mtDNA in the CSF from MS patients have identified increased amounts of cf-mtDNA copies in RRMS individuals with an acute inflammatory response compared to controls (Varhaug et al., 2017).

However, interestingly, Lowes et al. (2019) reported a reduction of CSF cf-mtDNA in post-mortem progressive MS (Lowes et al., 2019), while Leurs et al. (2018) observed increased levels of cf-mtDNA in the ventricular CSF of progressive MS compared to controls (Leurs et al., 2018). The discrepancy between these results may be because one study was performed with living subjects and the other post-mortem. Therefore, more detailed analysis and independent studies are needed to determine which changes in ccf-mtDNA levels may reflect MS progression, and whether it may indeed be a hallmark of broader neurodegeneration; these findings will play a key role in determining the value of circulating cf-mtDNA as a specific biomarker in neurodegenerative diseases.

## 3. Clinical assessment of neurodegeneration and progression of disability in MS–neuroimaging markers

While the EDSS is commonly used for assessing the clinical condition of MS patients in everyday medical practice, it appears to be less sensitive when detecting clinically-significant factors contributing to the progression of disability in SPMS patients, especially upper limb and cognitive dysfunctions (Cadavid et al., 2010). Instead, it has been recommended to use the EDSS-Plus scale to assess SPMS progression; this tool consists of a combination of the EDSS score, T25FW (Timed 25-Foot Walk Test), and 9HPT (9-Hole Peg Test) (Cadavid et al., 2017). The T25FW measures the time taken for a patient to walk a distance of 25 feet (7.5 m), while the 9HPT rates the patient’s ability to insert nine pegs in and out of holes in a box. The EDSS-Plus score (EDSS, T25FW, and 9HPT) is twice as sensitive as the EDSS alone in evaluating disability progression in SPMS patients (59.5 vs. 24.7%) (Bin Sawad et al., 2016; Cadavid et al., 2017). Alternatively, the Multiple Sclerosis Functional Composite (MSFC) assesses the ability to move over short distances (T25FW), upper extremity function (9HPT), and patient cognitive function, using the Paced Auditory Serial Addition Test (PASAT). However, the PASAT test, which assesses the auditory speed of processing information and counting skills, does not appear to be very sensitive when testing for cognitive function decline in SPMS (Orbach et al., 2012).

In addition, as patients can improve their 9HTP and PASAT test scores through repetition, the T25FW test seems to be the most reliable tool for the clinical assessment of disease progression (Rosti-Otajärvi et al., 2008). Despite physical disability, 40–65% of patients with progressive MS, experience cognitive impairment, which affects the quality of their life to a greater extent compared to physical impairment (Messinis et al., 2010). Cognitive impairment is also more closely related to cortical and subcortical atrophy; however, it is often difficult to measure using only the EDSS (Eijlers et al., 2018; Højsgaard Chow et al., 2018; Manca et al., 2019). Another diagnostic tool for the assessment of cognitive functions in MS patients, which also assesses the ability to concentrate, maintain attention and visual-motor speed, is the Symbol Digit Modalities Test (SDMT) (Kalb et al., 2018). The SDMT is more sensitive than the PASAT, and better at distinguishing RRMS from SPMS than various other neuropsychological assessments (López-Góngora et al., 2015; Ntoskou et al., 2018; Strober et al., 2019). However, a recent study suggests that SDMT scores improve with follow-up, possibly through repetition by patients, and thus does not accurately reflect the steady decline in cognitive function experienced by SPMS patients (Koch et al., 2021). Therefore, arguably the best diagnostic tool for assessing disease progression in patients with SPMS would be one that involves the use of several tests assessing deterioration in the clinical condition on various levels. However, the EDSS currently remains the most common scale in actual medical practice and is often the only one used.

The main method for diagnosing and radiologically monitoring the progression of MS and the efficacy of disease-modifying treatment (DMT) is MRI (Calvi et al., 2022). It exhibits high sensitivity in detecting white matter lesions (WMLs) across conventional T2-weighted sequences, proton density, fluid-attenuated inversion recovery (FLAIR), and T1-weighted (T1) sequences. While, WMLs are a pathological hallmark of MS, and the appearance of new lesions signifies recurrent clinical disease activity, sustained disability progression seems to be more closely linked with brain and spinal cord atrophy than to the emergence of novel WMLs. However, certain WMLs show persistent activity, which is also correlated with the clinical progression of the disease.

Four main subtypes of MS lesions have been identified: early active; chronically active (also known as mixed active/inactive, slow-expanding, or “smoldering”); inactive and remyelinated (sometimes termed “shadows”). New T2 lesions correspond to early active lesions and might exhibit transient T1 contrast enhancement during the initial stage, owing to gadolinium leakage, usually enduring for a span of 2–6 weeks. Subsequently, certain lesions undergo different processes: remyelination, inactivation, or persistent demyelination. Chronic black holes characterized by T1-sequence hypointensity lasting at least 6–12 months are related to pathologically greater myelin depletion and axonal density changes. Some of the radiological markers that seem to be most closely associated with the progression of MS and thus the transition to SPMS are discussed below.

### 3.1. Brain and spinal cord atrophy in MRI

Gray matter (GM) atrophy occurs in all types of MS and correlates with the progression of disability (Eshaghi et al., 2018). It is a well-established marker of neurodegeneration in MS when assessed by MRI volumetric methods (Sastre-Garriga et al., 2017). Loss of axons, and consequently loss of brain tissue, was previously considered mainly as the result of long-term inflammatory damage to myelin, a pathological process that could only be observed in the very late stages of MS (Sastre-Garriga et al., 2017). However, MRI studies performed in MS patients to assess the volume of brain tissue have found the degree of atrophy to significantly evolve over the short duration of the disease, and for it to correlate with the progression of clinical disability (Sastre-Garriga et al., 2017). It is, however, important to distinguish between the pathological atrophy of brain tissue associated with MS from physiological changes in mass. Based on previous studies, an annual change in brain volume of more than 0.4% was considered the cut-off point for pathological atrophy in MS; this was added to the criteria for disease inactivity, defined as the NEDA-4 index defined as “no evidence of disease activity” (De Stefano et al., 2016; Zivadinov et al., 2016a). Cortical atrophy appears to be more advanced in SPMS compared to RRMS (−0.87 vs. −0.48%, respectively) (Eijlers et al., 2019). Eshaghi et al. (2018) found that SPMS patients had the smallest initial volume of GM and deep gray matter (DGM) (Eshaghi et al., 2018). Comparing patients with SPMS and RRMS, significant differences in the volume of brain tissue were observed between patients with disability progression measured by the EDSS scale and those without (Sastre-Garriga et al., 2017). It was shown that the rates of temporal and occipital gray matter atrophy in SMPS (−1.21 and −1.24-%) were significantly faster than in RRMS (−0.76%), CIS (−0.75%) and (−0.63%), and in healthy controls (−0.51%) and (−0.23%); these differences were all significant (*p* < 0.05).

It has also been observed that some areas of the brain show earlier atrophy compared to others. These areas include the cingulate cortex, the GM of the insular and temporal cortex, and the DGM (putamen, caudate nucleus) (Steenwijk et al., 2016; Eshaghi et al., 2018). However, only the rate of GM loss in deeper structures was associated with disability progression (*p* < 0.001) (Eshaghi et al., 2018). Moreover, patterns of cortical atrophy appear to be more strongly associated with the progression of clinical dysfunctions, especially cognitive functions than global cortical atrophy (Steenwijk et al., 2016).

Another volumetric measure of neurodegeneration in MS assessed in MRI is the volume of the thalamus. Baseline thalamic atrophy is associated with a higher risk of an increase in EDSS over 5 years, as well as a lower chance of achieving “no evidence of disease activity” (NEDA-3) after 2 years (Hänninen et al., 2019, 2020). Anterior thalamic nucleus atrophy correlates with diminished cognitive processing speed (Bergsland et al., 2016). In turn, corpus callosum atrophy emerges as among the most sensitive MRI indicator for memory and processing speed (Papathanasiou et al., 2017).

Another indicator of the progression of neurodegeneration in SPMS seems to be the volume of the ventricles of the brain. Numerous studies have shown that an increase in the amount of CSF in the ventricles of the brain visible in MRI is an important predictor of disability progression in MS from early clinical stages (Zivadinov et al., 2016a). In a decade-long follow-up involving 181 patients with RRMS, those exhibiting progressive disability manifested a 41% increase in ventricular CSF volume, whereas the stable group demonstrated a 26% increase. Alterations in ventricular CSF volume showed a robust association with GM changes and cortical atrophy, indicating that brain ventricular volume may be a major measure of brain tissue atrophy reflecting changes in brain and GM volume.

**FIGURE 1:**
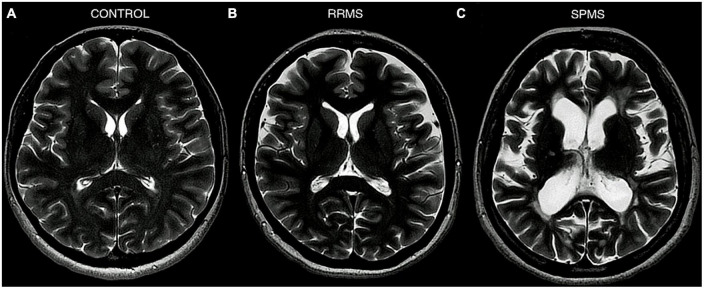
MRI T2-weighted sequence, axial section. Comparison of the degree of brain atrophy and brain ventricular volume between healthy controls **(A)**, patients with RRMS **(B)**, and patients with SPMS **(C)**. Material from the resources of the Medical Radiology Department of the Military Medical Institute–National Research Institute.

A comparison of the degree of brain atrophy and brain ventricular volume between a healthy control, a patient with RRMS, and a patient with SPMS is presented in Figure 1. All patients are under the care of the Neurological Clinic of the Military Medical Institute–National Research Institute.

There are several external confounding factors to consider when analyzing brain atrophy, including hydration changes, circadian fluctuations (brain volume is larger in the morning), lifestyle (smoking, alcohol consumption), menstrual cycle (spikes in brain tissue during menstruation), and comorbidities (hypertension, hyperlipidemia, and heart disease increase atrophy) (Duning et al., 2005; Enzinger et al., 2005; De Stefano et al., 2014; Nakamura et al., 2015; Zivadinov et al., 2016b). Furthermore, while inflammation can induce a transient increase in brain volume in the short term, DMTs can diminish swelling, thereby triggering an accelerated, non-tissue-related decline in brain volume termed “pseudoatrophy” (Zivadinov et al., 2008). In addition to confounding factors, several technical impediments hinder the integration of atrophy assessment with clinical practice. These encompass diversity in acquisition protocol, distortion differences, and fluctuations attributed to motion and scanner discrepancies (Zivadinov et al., 2016a; Sastre-Garriga et al., 2020).

An important predictor of disability progression in patients with MS is spinal cord atrophy (SCA) in the cervical region (Schlaeger et al., 2014). Spinal cord MRI assessment is performed at the onset of the first symptoms in two groups of patients: those with a clinical picture suggesting spinal cord involvement, and those with CIS who do not fully meet the criteria for spatial dissemination (Rovira et al., 2015). Association between SCA, disability progression, and motor dysfunction occurs autonomously from brain atrophy (Sastre-Garriga et al., 2020). A recently published meta-analysis confirmed the relationship between SCA and the clinical progression of MS measured by the EDSS (Song et al., 2020). Subsequent studies have shown that the volume of the spinal cord varies depending on the subtype of MS, with the cross-sectional studies recording the deepest atrophy in patients with progressive MS (Sastre-Garriga et al., 2020). By comparing the cross-sectional area (CSA) of the spinal cord, RRMS can be distinguished from progressive types of MS (*p* < 0.001) (Casserly et al., 2018). Furthermore, SCA progresses faster in patients who demonstrate disease progression after 2 years (Lukas et al., 2015). This is a very sensitive biomarker, especially since the estimated annual incidence of SCA is higher than that of brain atrophy in MS patients (−1.78 vs. −0.5%) (Casserly et al., 2018; Moccia et al., 2019). Elevated rates of cervical cord area loss correlated with disability progression, irrespective of concurrent clinical and radiological parameters, including the occurrence of spinal demyelination lesions. However, due to the anatomical (greater mobility, smaller dimensions) and imaging (lower tissue contrast) features of the spinal cord, it is technically more difficult to assess SCA than brain tissue atrophy.

### 3.2. Slowly expanding lesions (SELs)

In MS, during the early development phase of RRMS, some demyelinating lesions are remyelinated at an early stage, transforming into “shadow demyelinating plaques,” which prevent axonal degeneration (Goldschmidt et al., 2009). However, some of the lesions transform into smoldering plaques or develop into slowly expanding lesions (SELs) (Prineas et al., 2001; Kutzelnigg et al., 2005). Chronic active peripheral iron-containing lesions, i.e., the SELs, comprise a subgroup of MS lesions with inactive demyelinating centers that additionally maintain or develop continuous periphery myelin disintegration. They expand toward the surrounding white matter, unlike the non-ferrous lesions, which shrink significantly over time (Calvi et al., 2022). These represent one of the most common types of lesions observed in histopathological examinations of patients with PPMS and SPMS.

Histopathologically, SELs have a central area with only low numbers of macrophages, or none, surrounded by an iron rim with a characteristic pro-inflammatory phenotype. The lesions seem to be more destructive and hence appear as hyperintense in T1-weighted images (black holes) due to the greater reduction of myelin and axonal density (Dal-Bianco et al., 2021). In addition, it has been noted that the number of chronic enlarging lesions (SELs) correlates with brain atrophy. Patients with multiple SELs (≥4 SELs) are characterized by higher radiological disease activity (greater burden of lesions and increase in ventricular volume, smaller volume of the brain and basal ganglia), higher level of motor disability (EDSS) and cognitive disability (SDMT, PASAT) as well as a transition to the progressive form of the disease at a younger age (Luchetti et al., 2018; Absinta et al., 2019). Moreover, as SELs appear to have a good image-pathological correlation, they seem to be good radiological markers for assessing disease progression and even conversion to SPMS.

### 3.3. Iron rim lesions

Since SELs can be distinguished from lesions with higher remyelination potential based on the accumulation of iron at their edges, MRI SWI-based iron rim imaging may be a useful radiological marker for progressive MS. Previous histological studies have confirmed the presence of iron deposits in chronic, active demyelinating MS lesions (Mehta et al., 2013). Although the molecular basis of iron accumulation in MS lesions remains evasive, several possible mechanisms have been proposed, such as an increase in iron-rich oligodendrocyte debris, iron sequestration of macrophages and microglia, and products of local microhemorrhage following venous wall injury (Williams et al., 2012).

Maximum iron accumulation was present on the edges of reactive slow-expanding chronic MS lesions, where oligodendrocytes undergo varying degrees of destruction (Chawla et al., 2016). Similarly, microglia and iron-containing macrophages, which are located mainly at the rims of chronic reactive lesions, are also subject to dystrophy, resulting in varying levels of iron being deposited across different ranges of demyelinating plaques (Mehta et al., 2013). Dal-Bianco et al. (2017) report that lesions with an iron rim significantly expanded over 3.5 years compared to those without a rim, which tended to shrink (*p* = 0.003) (Bagnato et al., 2011); in addition, these changes were seen in RRMS and SPMS patients but not in optic neuritis patients.

**FIGURE 2:**
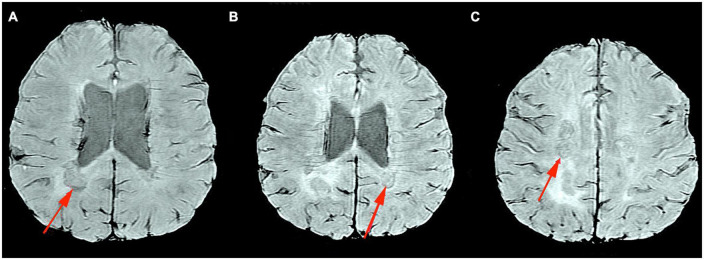
MRI Swan sequence, axial section. Iron rim sign in a patient with SPMS **(A–C)**. Material from the resources of the Medical Radiology Department of the Military Medical Institute–National Research Institute.

In conclusion, pro-inflammatory macrophages/microglia in SELs may or may not contain iron deposits, while the presence of specific iron-rich rims is a strong indicator of the pro-inflammatory status of microglia or macrophages. Furthermore, no iron rims were observed around most of the “plaque shadows”; this may indicate that remyelination is limited to lesions that never underwent rim iron accumulation. These findings suggest that the occurrence of iron rims in MRI images of the brains of MS patients could indicate progressive tissue deterioration. In addition, the presence of a higher number of iron-margined lesions is associated with more frequent clinical relapses and their association with clinical disability suggests that they might also constitute a risk factor for clinically progressive disease development. An MRI image, SWAN sequence, of a typical iron rim in a patient with SPMS is presented in Figure 2; the patient is under the care of the Neurological Clinic of the Military Medical Institute–National Research Institute.

### 3.4. Leptomeningeal contrast enhancement (LMCE)

In addition to brain tissue volume measurements, which generally detect irreversible brain atrophy and thus constitute a late marker of disease, researchers are still trying to identify imaging markers of early pathophysiological processes preceding neuroaxonal pathology in the cerebral cortex that may underlie progressive MS. A promising area that may be useful for predicting neurodegeneration concerns changes in leptomeningeal inflammation (Dal-Bianco et al., 2017). Clusters of inflammatory cells within the meninges that persist over a long period can sustain a myelin-specific immune response and thus directly contribute to subcortical demyelination and neurodegeneration. Histopathological studies have revealed the presence of perivascular lymphocytic and mononuclear infiltrates in the Gd+ (gadolinium) enhancement areas associated with cortical demyelination. The relationship between ectopic lymphocytic aggregates of the meninges and pathology of the cerebral cortex was first demonstrated by Serafini et al. (2004), who report that immunostaining confirmed the presence of massive B-cell infiltration in such aggregates (Serafini et al., 2004). Reich et al. (2014), report that 40 out of 240 cases of MS patients (17%) showed small lesions with enhanced contrast in the meninges, compared to 0/10 of healthy volunteers (*p* = 0.37) (Reich et al., 2014).

Leptomeningeal contrast enhancement (LMCE) was localized rather than diffused, and often occurred in the sulcus. Amplification was observed twice as often in SPMS (23/97 cases, 24%) as in RRMS (17/143, 12%). In addition, once detected, the enhancement remained stable over time, regardless of the DMTs administration. LMCE was neither associated with contrast-enhancing active WM lesions, nor with CSF OCBs, immunoglobulin G index, age, sex, or disease duration.

Absinta et al. (2015) identified focal contrast enhancement within the meninges of 74 out of 299 MS patients (25%) compared to only one out of a group of 37 healthy controls (2.7%; *p* = 0.001) (Howell et al., 2011). Enhancement was almost twice as common (*p* = 0.009) in SPMS (39/118 cases, 33%) than in RRMS (35/181, 19%). Likewise, it was noted that the enhancing foci mostly remained stable throughout the evaluation period (up to 5.5 years). Also in the meta-analysis by Ineichen et al. (2022), it was shown that the presence of LMCE was statistically significantly less frequent in RRMS compared to the progressive form of MS (Absinta et al., 2015).

In a study of 54 MS patients, Makshakov et al. (2017) compared the presence of LMCE, identified by 3-Tesla MRI, FLAIR sequence, the EDSS score, the number of clinical relapses during the 5-year course of the disease, and the presence of contrast-enhancing foci in T1-weighted MRI images. LMCE was identified in 41% (22/54) of patients and linked to extending disease duration (*p* = 0.0098), elevated EDSS score (*p* = 0.039) but not with increased relapse rate (*p* = 0.091). However, no relationship was noted between a higher number of LMCEs and the presence of contrast-enhancing lesions in the T1-weighted images (*p* = 0.384). The analysis adjusted for age, sex, and disease duration showed that LMCE had a significant effect on cortical volume (*p* = 0.043), total GM volume (*p* = 0.043), and cerebral ventricular volume (*p* = 0.039) (Makshakov et al., 2017).

**FIGURE 3:**
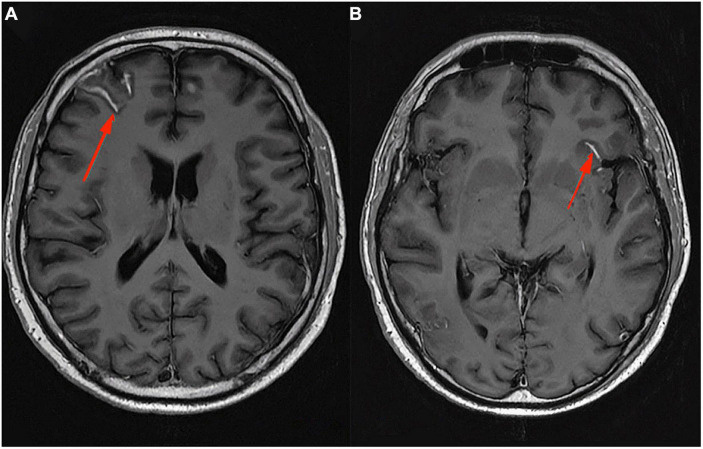
MRI T1-weighted sequence with contrast, axial section. Leptomeningeal contrast enhancement in a patient with SPMS **(A,B)**. Material from the resources of the Medical Radiology Department of the Military Medical Institute–National Research Institute.

Following Zivadinov et al. (2017), it is hypothesized that LMCEs may be associated with greater brain atrophy, in particular GM and cortical atrophy (Zivadinov et al., 2017). However, the presence of LMCE was not found to be associated with a higher risk of relapses or focal enhancement after contrast in T1-weighted images. This suggests that the development of atrophy has a different origin, most likely neurodegenerative. Although leptomeningitis is often visible as a gadolinium enhancement on T2-FLAIR MRI, imaging of meningitis has proven technically challenging. Numerous blood vessels in the meninges strongly potentiate under the influence of gadolinium on T1-weighted images and are therefore difficult to distinguish from inflammatory foci. In addition, contrast material leaking into inflamed foci near the surface of the brain may mix freely with the CSF and thus may not reach sufficient concentrations to be visualized in MRI. The detection of meningeal pathology in MS is also complicated by the small size of meningeal infiltrates, which is usually less than 1 mm (Ineichen et al., 2022). LMCE in patients with SPMS is presented in Figure 3. The patient is under the care of the Neurological Clinic of the Military Medical Institute–National Research Institute.

### 3.5. PET 8 kDa translocator protein

Using radiolabeled ligands that bind specific targets, positron emission tomography (PET) imaging can detect chronic MS lesions with ongoing inflammatory activity (Calvi et al., 2022). PET radioligands bind to the translocator protein (TSPO) expressed by microglia/macrophages in diverse neurological conditions (Bergsland et al., 2019). In MS, histopathological studies found TSPO uptake to be a marker for chronically-active lesions and to be associated with those presenting an enhancing halo in MRI (Guilarte, 2019). Lesions with a strengthening rim demonstrated higher TSPO uptake than those without. In addition, such rims were noted in chronically-active lesions with high pro-inflammatory activity and iron deposition levels; in contrast, inactive lesions demonstrated low TSPO expression. It is possible that such chronic lesions can be identified as SELs.

Sucksdorff et al. (2020) found elevated TPSO uptake in WM demyelinating lesions as a predictive indicator of subsequent disability progression over a 4-year follow-up, irrespective of concurrent relapse activity (Sucksdorff et al., 2020). This implies that the heightened TPSO uptake phenotype of innate immune cells is deleterious, fostering extensive diffuse axonal impairment and the emergence of covert, gradual disease progression, regardless of activity recurrence. Importantly, this study confirms that augmented TSPO binding is an adverse event predictive of accelerated MS disease progression (Sucksdorff et al., 2020). Despite the promising results of this study, it should be noted that technical challenges and radiation exposure prevent the widespread use of TSPO-PET as a marker in clinical practice. As such, there is a need to identify markers related to innate activity cell activations, which are easier to measure.

**FIGURE 4:**
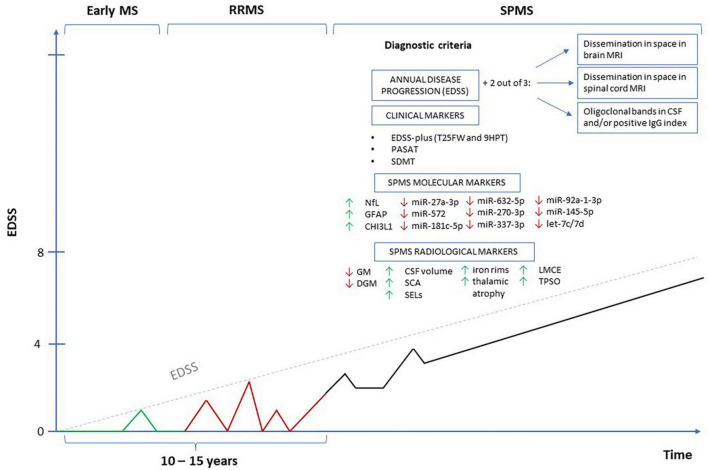
The diagnostic procedure schema for identifying the conversion of MS to SPMS, taking into account the clinical engagement of the disease (annual disease progression measured on the EDSS scale), radiological progression (defined as spatial dissemination), and incorporating the newly described diagnostic markers in the diagnostic process (both clinical, biochemical, and radiological), is presented. The entirety of the schema also encompasses the visualization of disease progression measured on the EDSS scale over the course of time (horizontal time axis).

In order to encapsulate all the described clinical and biochemical biomarkers, the proposed diagnostic procedural framework for the identification of the MS transformation to the progressive stage of the disease is delineated in Figure 4.

## 4. Further directions and conclusion

There is a pressing need to establish official criteria for the diagnosis of SPMS to improve current and future treatment options. Therapy must be introduced at the earliest possible stage of lesion progression to protect the CNS from the lasting effects of neurodegeneration. Accurate diagnostic tools to help predict the transition from RRMS to SPMS may play a key role in facilitating the early mitigation of the long-term effects of SPMS development. Fortunately, many factors are under intensive investigation as potential neurodegenerative markers, thus providing new opportunities to update diagnostic criteria; as such, new possibilities for their use should become apparent in the future. Modern technologies or the implementation of artificial intelligence algorithms may play important roles in determining the disease phenotype and in the application of targeted MS therapy.

Current data indicates that both innate and adaptive immune-mediated inflammation influence the early development and progression of MS. To date, the diagnosis of MS progression is based on retrospective clinical and radiological analysis, including worsening of the health of the patients and neurodegeneration. However, the day-to-day variation of neurological symptoms could hinder any objective detection of new symptoms or progression by neurologists. Therefore, future research should focus on evaluating whether the mentioned markers or a combination of the two, could serve as more sensitive tools to assess disease progression. If these markers’ discriminative potential is affirmed through autonomous cross-sectional investigations, the subsequent endeavor would entail enhancing the precision in appraising MS progression more effectively, leading to initiating as soon as possible appropriate treatment.

## Author contributions

AP-W, AD, KM, AS, and JS wrote the final version of the manuscript. AP-W, MD, and AS prepared MRI images (figures). AD and KM prepared the table. AP-W, AS, and JS supervised the manuscript. All authors read and approved the final draft of the manuscript and agree to be personally accountable for their own contributions.
